# Acquired Dyschromatopsia and Its Link to Drug Toxicity

**DOI:** 10.7759/cureus.76190

**Published:** 2024-12-22

**Authors:** Ahmed Ageed, Mohammed Dauwood Aslam, Sumayya El Haouari

**Affiliations:** 1 Medicine and Surgery, University Hospitals of Leicester NHS Trust, Leicester, GBR; 2 General Practice, Health Education West Yorkshire, Huddersfield, GBR; 3 Optometric - Glaucoma, Leicester Royal Infirmary, Leicester, GBR

**Keywords:** colour vision, colour vision defects, dichromatic, drug toxicity, dyschromatopsia, monochromatic, trichromatic

## Abstract

Colour vision defects (CVDs) can be both congenital and acquired, with acquired dyschromatopsia often associated with medication toxicity. This review explores various standardised colour vision tests used to detect these defects, including the Ishihara plate test, Farnsworth-Munsell 100 hue test, and anomaloscopes. These methods are evaluated for their effectiveness in diagnosing CVDs, particularly in acquired conditions. Specific focus is given to medications known to induce dyschromatopsia, including chloroquine and hydroxychloroquine (CQ/HCQ), digoxin, ethambutol (EMB), and phosphodiesterase-5 (PDE-5) inhibitors. CQ/HCQ primarily causes tritan defects at early stages of retinal toxicity and progresses to red-green defects with more advanced retinopathy. Digoxin-induced CVDs are typically temporary red-green defects linked to the inhibition of Na+/K+ ATPase in retinal cells. EMB, used to treat tuberculosis, is associated with blue-yellow (tritan) dyschromatopsia, likely due to optic neuropathy. PDE-5 inhibitors, such as sildenafil, cause transient blue-tinted vision due to their effect on the phototransduction cascade in cones. Although most drug-induced CVDs are reversible upon discontinuation, CQ/HCQ toxicity often leads to irreversible damage. This review underscores the importance of colour vision monitoring in patients on long-term medication therapy to detect early toxicity and prevent irreversible damage.

## Introduction and background

Colour vision is the capability of any organism to be able to distinguish objects based on the wavelength of light reflected, emitted, or transmitted [1]. Dyschromatopsia is simply defined as an impairment or defect of colour vision, commonly proportional to a degree of visual impairment [2], although this is not always the case.

Dyschromatopsia is usually separated into congenital and acquired. Acquired is much more prevalent than congenital dyschromatopsia [3], but the exact prevalence is unknown. It is difficult to distinguish whether an individual’s colour vision defect (CVD) is congenital or acquired unless they have been monitored previously or have had the CVD induced. There are several major causes of acquired CVDs; not all the associations are fully explained. They include glaucoma, cataracts, optic neuritis/neuropathy, photoreceptor disorders, retinal/chorioretinal dystrophies, age-related macular degeneration (AMD), fundus detachments, cerebral achromatopsia, and environmental dyschromatopsia [3]. However, the focus of this review will be acquired dyschromatopsia and its connection to these specific drugs: chloroquine/hydroxychloroquine (CQ/HCQ), digoxin, ethambutol (EMB), and phosphodiesterase-5 inhibitors (PDE-5 inhibitors).

People with normal colour vision (normal trichromats) have fully functioning cone photoreceptors of all three types: long, medium, and short [4]. This enables the full spectrum of colours within the visible range to be seen, including all wavelengths of light between 380 and 700 nm [5]. A CVD arises when one or more cones are missing or possess an abnormal function [1].

Congenital CVDs (CCVDs) result from non-pathological photopigment level changes that remain the same over time [1]. It is a recessive X-linked disorder, where total colour blindness is rare and partial is much more common [6], present from birth or childhood. The prevalence of CCVDs is greater in males (8%) than in females (0.5%) worldwide [7].

Several studies show that the prevalence of acquired dyschromatopsia is considerably higher than believed [8]. One study suggested 2% [9], but the exact amount is currently unknown. The most common form of CCVD is one that affects long- and medium-wavelength photopigments (red-green) [10], whereas tritanopia/tritanomalous (tritan, blue-yellow) defects are more common in acquired CVDs.

## Review

Standard colour vision tests 

There are multiple ways that colour vision can be detected, assessed, and monitored.

The screening test found in nearly all optometric practices is the pseudoisochromatic Ishihara plate test [11]. There have been studies showing that the test is not sensitive enough to detect acquired dyschromatopsia but is better for CCVDs [12]. This is due to acquired CVD usually being S-cone mechanism deficient rather than M-L cone deficient, unlike CCVD [3]. The American Optical Hardy-Rand-Rittler (AO-HRR) test is also primarily used for CCVD (including tritan) detection but can detect moderate and severe acquired CVDs [13]. It consists of various colour difference steps to grade the defect. The FM-100 hue test is another ordering test which consists of the patient grading the progression of 100 disks of Munsell paper [14]. It is considered the gold standard in terms of acquired CVD detection and diagnosis [15]. The D-15 test is also an ordering colour vision test consisting of coloured caps (similar to FM-100 hue but fewer), and the aim is to dichotomise the colour normal from the colour deficient patient [16]. However, both FM-100 hue and D-15 are not good screening tests as there are too many requirements and the length of time needed to complete them is much longer than Ishihara, which remains the most practical for use in a clinical setting.

There are desaturated versions that help to improve the sensitivity of the testing. Another form of colour vision testing is by matching, and the primary one to look at would be Rayleigh. Lord Rayleigh developed an equation to be used for patients to match a red-green mixture to a monochromatic yellow [17], which is used in anomaloscopes.

These tests have been used to assess and diagnose the colour vision associated with the drugs that will be spoken about in this review.

Chloroquine and hydroxychloroquine (CQ and HCQ)

CQ is a prototypical antimalarial agent used to prevent and treat malaria and amebiasis (intestine infection) [18,19]. HCQ is a derivative of CQ but is less toxic. Its main purposes include the treatment of systemic lupus erythematosus (SLE), rheumatoid arthritis (RA), juvenile idiopathic arthritis (JIA), and Sjörgen’s syndrome [20]. Studies have shown that the main connection between CQ and HCQ with dyschromatopsia is CQ/HCQ retinopathy and optic neuropathy. CVDs are often overlooked as an early sign of retinal dysfunction despite retinal toxicity frequently being characterised by defective colour vision [21]. One study stated that the tritan defect is the most prevalent form of dyschromatopsia associated with CQ/HCQ toxicity [19]. On the other hand, with further increased toxicity, a red-green defect is seen.

The full mechanism of CQ/HCQ and how they can affect colour vision is not fully understood. The molecule binds to melanin compounds in the retinal pigment epithelium (RPE), causing these compounds to accumulate. Over time, this accumulation disrupts the normal metabolism of the RPE, ultimately leading to toxicity [22]. The bull’s eye maculopathy is caused by the damaged RPE moving to damaged areas where the photoreceptors have shed outer segments), leading to photoreceptor loss at the macula. This could explain the CVD caused at the bull’s eye maculopathy stage of CQ/HCQ toxicity, as there is a loss of cones.

One clinical study looked into the effect that CQ/HCQ has on the inner retinal structures [23]. They separated the subjects into three groups: group I, consisting of four with CQ/HCQ toxicity with abnormal fundus; group II, consisting of eight with chronic exposure without fundus changes; and group III, consisting of eight controls. At the end of the experiment, only three of the patients in group I, all with bull’s eye maculopathy, had an abnormal colour vision with the pseudometric Ishihara plate test. The third patient had one eye with CVD, but the other eye was normal. This shows that CVD induced by CQ/HCQ may not be binocular and may affect one eye only, which was not assessed by many studies and is a strong point in this particular study. The sample size ended up being small and, therefore, lacked the ability to represent a general population. This would be needed to decide if the ocular toxicity had a significant connection with forming CVDs.

A study conducted by Missner et al. also evaluated the use of colour vision assessment in order to measure the progression of CQ/HCQ treatment and toxicity [24]. Twenty patients, all under long-term treatment of CQ and HCQ, had their colour vision observed. D-15 and AO-HRR were used, with 15 patients under D-15 and six patients under AO-HRR having a form of CVD detected. The form of CVD was not categorised. There was high variability in the results of the colour vision testing, so the observers suggested the multifocal electroretinography (mfERG) technique as more effective. Another study completed by Vu et al. looked specifically at the detection of CVDs in patients with CQ retinopathy and included a wider range of colour vision tests [25]. Thirty patients were monitored, all with CQ retinopathy, and 28 failed at least one of the colour vision tests. The tests included Ishihara, desaturated D-15 (Dsat-15), AO-HRR, and the City University test (CU). The variety of tests had different levels of specificity and sensitivity, so it did allow for a better evaluation of colour vision to be made. They concluded that there is a significant correlation between CQ and acquired dyschromatopsia. However, in both studies, the form of acquired dyschromatopsia was not noted.

Cabral et al. [18] conducted a study with patients on the long-term use of CQ or HCQ to treat SLE and RA. Colour vision monitoring was only observed in nine of the 217 patients with advanced-stage maculopathy. Only one patient had significantly altered colour vision (seen on Ishihara), and only abnormal results came from patients with grave maculopathy. The limitations should have been much smaller; however this, was not the case. Firstly, the type of CVD was not stated, which is key to finding out the specific impact CQ/HCQ has on colour vision. It only included nine patients being colour vision monitored, and the least effective colour vision test, Ishihara, was used, which does not pick up acquired CVDs effectively unless quite severe. The study should have had a larger population sample size and used more effective colour vision tests in order for appropriate comparisons to be made (i.e. FM-100 hue).

All studies made sure not to include patients with a previous history of CVDs, and the majority did not include patients with any other ocular conditions that could have an impact on the results. It was agreed upon by all the studies that the severity of induced drug toxicity impacted the degree of dyschromatopsia that was experienced by patients. Patients who were on CQ/HCQ, but without any retinopathy/maculopathy, did not experience a CVD, and this was statistically insignificant. However, the majority of patients who had bull’s eye maculopathy and severe toxic retinopathy did have an acquired CVD due to the ocular toxicity of the CQ/HCQ treatment. This is one of the drugs mentioned where colour vision does not return, even after the drug has been withdrawn from the system [19]. This is due to the compound of the drug remaining in the retina for several years, even after treatment discontinuation [26].

Digoxin

Digoxin comes under the digitalis family of drugs, which also includes digitoxin, and is extracted from the leaves of the foxglove plant Digitalis lanata [27,28]. Digoxin is a form of anti-dysrhythmic that is usually prescribed to control heart failure and cardiac arrhythmias [19] and to relieve symptoms. It decreases heart failure patient hospitalisation and controls the increased ventricular rate found in patients with atrial fibrillation [29]. Around 80% of patients, even on therapeutic levels of digoxin, develop a CVD [30]. Digoxin visual toxicity is suspicious when there are visual symptoms, and the digoxin level is higher than the therapeutic level [31], even though non-toxic levels can produce ocular toxicity symptoms as well [32].

The retina is considered the initial site of toxicity. The exact mechanism of how it impacts visual function is not fully understood. Its primary function is to inhibit the Na+ /K+ ATPase pump. This leads to an increase in sodium ions (Na+) in cells, which then leads to an increase in calcium ions (Ca2+), inevitably increasing heart contraction. The same enzymes are found in the cell membranes of photoreceptors, Müller cells and the RPE [33-35]. This could be the connection between the digoxin mechanism and colour vision.

Patients under digoxin do usually complain of colour vision defects and degeneration. Be that as it may, colour vision testing to be used as an indicator for digoxin toxicity has been overlooked in the past [36]. Dyschromatopsia is not a usual sign in patients who have been under long-term digoxin treatment [30,37]. This is a result of the dyschromatopsia that is induced by the digoxin, usually being seen as an R-G defect that is temporary, with possible recovery occurring after the drug has been withdrawn [18].

Chuman et al. conducted a study on two patients with digoxin toxicity [38]. Patient 1 described vision as appearing whiter, while patient 2 described colours as appearing more faded. As well as the Ishihara and AO-HRR, the FM-100 hue test was used, which is the golden standard for colour vision testing. This meant test results would be accurate. As the first patient’s digoxin serum levels decreased, the colour vision errors made decreased on the FM-100 hue. This was also true for the second patient being monitored as well. They also observed that both B-Y and R-G colour defects were observed and that withdrawal of the drug led to major improvements in colour vision. However, this study was conducted over 20 years ago, and there have been updates in the way these types of patients would have been treated, particularly with them both having cataract. Additionally, the sample size would have been very inaccurate to extrapolate against a larger population size.

A survey was completed, testing 30 patients who all received digoxin, which was compared with a control group in terms of colour vision tests [39]. The two types of CVD that were assessed were the red-green defect and the tritanopia defect using Ishihara, HRR R-G, and tritan plates: city Tritan and Lanthony Tritan Album. They demonstrate that there is not a significant difference between the control group and the digoxin group in terms of slight CVDs, whereas there was a major difference in severe tritan defects. The tritan errors were common in the controls,, but they were only slight or occasionally moderate (in the Lanthony Album test). More severe CVDs were noticeable with the patients under digoxin and more likely tritan. The results did not demonstrate digoxin toxicity as an indicator of CVD because 27 of the patients were still within the therapeutic window that acquired dyschromatopsia.

All studies confirmed that there is a significant acquired dyschromatopsia associated with digoxin toxicity. Even though therapeutic levels of digoxin did show some CVDs, they were not as significant. A patient reported “90% improvement” in visual symptoms after discontinuation of digoxin [28], so this confirms the fact that, once the drug is out of the system and not being used anymore, dyschromatopsia will also leave. However, the lack of recent papers and small sample size means that more needs to be looked into for digoxin toxicity and its connection to dyschromatopsia.

Ethambutol (EMB) 

EMB is known to be the most universally used treatment for TB, as well as non-tubercular mycobacterial diseases. This is mostly due to it being the least toxic antituberculosis agent with the lowest incidence of ocular toxicity [40,41]. For a long time, there has been a connection between the use of EMB for TB treatment and the possibility of acquired dyschromatopsia. Garg et al. conducted an evaluation in India, showing that 12.3% of those receiving anti-tubercular medications gained CVDs in their eyes [42]. Most studies have shown that there is a predominant effect in ascertaining B-Y defect, which is subtle and can only be detected with the desaturated panel of Lanthony [43], whereas some other studies have pointed out that there is a predominant R-G dyschromatopsia seen in patients [44]. Even colour vision tests such as Ishihara plates and D-15 can elicit the CVD [42].

The EMB ocular neurotoxic effect mechanism is not exactly understood. There have been various hypotheses for EMB’s toxicity connected to its zinc-chelating effect [45-47] and its effect on the excitotoxicity pathway [48]. EMB is a metal chelator [49], and its actions include inhibiting arabinosyltransferase (in mycobacterial cell-wall synthesis) and disrupting oxidative phosphorylation and mitochondrial function, which leads to cellular apoptosis and tissue injury [45]. Two studies also showed that there is a possibility that EMB actually affects the amacrine and bipolar cells as colour vision is affected, but visual acuity is not [50,51]. There have been some animal studies that show this, where the retinal ganglion cells (RGCs) were damaged in monkeys with EMB-induced optic neuropathy [52].

Kaimbo et al. completed a two-year and eight-month study assessing colour vision with TB patients in Congo who had no previous CVDs (tested using Ishihara plates) [53]. This was good as they excluded any CCVD patients and the results were only due to drug-induced acquired dyschromatopsia. The results confirmed the current outlook that colour vision could be used to monitor the progression of EMB complications. This is agreed with by Polak et al. [44] who stated that dyschromatopsia is potentially the most sensitive detector of early EMB optic neuropathy/neuritis. This side effect is of most concern to ophthalmologists [49,53]. Only after five months of EMB treatment were the colour vision disturbances (linked to ocular toxicity) detected. Overall, a majority of patients were asymptomatic; however, results show one patient failing AO-HRR, three patients with tritanopia (B-Y defect) on the D-15 test, and 15 patients with high total error scores on FM-100 hue test. There were three patients during the follow-up period that gained complete colour blindness. Treatment was stopped, and of the three, two regained colour vision, but the third patient showed no improvement in colour vision nor visual acuity.

Another study was completed on 64 newly diagnosed TB Filipino patients (no colour vision abnormalities) treated with directly observed treatment short (DOTS) course [54]. DOTS is a form of TB treatment that is known for being cost-effective and quickly makes infectious disease non-infectious [55]. After the first month of treatment, colour vision was measured: Ishihara results showed all patients as normal, one patient failed D-15, and 30 failed Lanthony desaturated (10 with tritan and 20 unclassified). Any CVDs that were noted did revert back to normal after an average of 37.85 days, showing the non-permanent nature of the dyschromatopsia with EMB toxicity. Despite normal visual acuities throughout the testing period, some patients complained of colour vision disturbances. This also agrees with the arguments presented, suggesting that monitoring CVDs with patients receiving EMB could be a good way to detect early EMB toxic optic neuropathy.

In the study conducted by Kaimbo et al., the wide variety of colour vision tests allowed for appropriate analysis to be made, which included Ishihara plates, AO-HRR, FM-100 hue, and D-15 [53]. They all vary in specificity and selectivity that proved effective for comparisons to be made. There were several limitations to this study that could have affected the reliability of the results. One fault was that there was no mention of the length of time it took for the patients to regain colour vision. The second issue is the wide age range; this is a significant factor in the progression of a CVD, namely, if the older patients have cataracts. The study did not say when the colour vision was regained and the test was only conducted on one of the subject’s eyes. However, acquired CVDs may be present in one eye only, so this study may have missed this. Similar with Cruz et al.'s study, the subject age was not a controlled factor, and it was determined that age was the only significant risk factor for dyschromatopsia with the EMB treatment [54].

In summary, both studies conclude that EMB causes acquired dyschromatopsia, and once the therapy is removed, all the patients recovered to normal colour vision. Regardless of the concentration of EMB, the effect remained the same. Both studies included a wide age range,, and this could have also affected the results, as acquired dyschromatopsia was significantly linked to patients' age. It is recommended to have smaller age ranges per study, or within each study, the patients are separated by age to make adequate comparisons. The form of dyschromatopsia linked to EMB ocular toxicity was tritanopia. Results also show that the Ishihara test was an inappropriate colour vision testing tool to use.

Phosphodiesterase-5 inhibitors (PDE-5 inhibitors)

The most well-known PDE-5 inhibitor is sildenafil (Viagra®), but there is also vardenafil (Levitra) and tadalafil (Cialis). PDE-5 inhibitors are mainly used for erectile dysfunction and are frequently prescribed to older men [56]; they increase smooth muscle blood flow [57]. Tait et al. [19] described the PDE-5 inhibitor as selectively inhibiting cyclic guanosine monophosphate (cGMP). PDE-6 (present in rod and cone photoreceptors) is also inhibited with an efficacy of approximately 10% of PDE-5 [57], which has been shown to play a significant role in the colour vision defect mechanism. PDE-6 is part of the phototransduction cascade reducing cGMP that leads to hyperpolarisation of the cell. PDE-5 inhibitors break this process, and this is what leads to short-term changes in the rod/cone function.

It has been reported that PDE-5 inhibitors can cause “bluish vision” as users have trouble discriminating purple, blue, and green [56]. There has been a lot of controversy in terms of whether the literature agrees about PDE-5 inhibitors’ contribution to CVDs. For example, one study [58] concluded that PDE-5 inhibitors do not show a significant change in colour vision comparable to the general population. Another study concluded that none of the five volunteers complained of visual disturbances, and there was no significant effect on colour vision [59]. Tait et al. [19] described the colour vision defects as transient and no consistency, indicating that there was no effect on the ocular structures, including the retina, leading to no long-term effect on colour vision. Meanwhile, in another study, it conveys the opposite; that is, there were significant alterations in colour vision [60].

Luu et al. [61] studies 18 healthy normal trichromatic individuals (male and female), 14 were given sildenafil and the rest water. They were tested before and after with a desaturated D-15 test. Only one of the patients reported blue-tinted vision, and another one reported red and blue speckled vision. There was a statistically significant difference in total errors made by both eyes in 10 of the 14 sildenafil patients. This was mostly between similar colours; however, three patients did show R-G confusion. One concern observed was the possibility of the results being affected by tiredness or distraction, as the desaturated D-15 test requires concentration. The test was repeated one hour before and after the drug was given to the patients, which could have increased this impact on results. One contraindication of this is the fact that the desaturated D-15 sets a higher level of difficulty. This improves the accuracy, meaning anyone who was proven to have a CVD but passed the test would show a milder deficiency.

Stockman et al. [57] also conducted a study using a conventional Maxwellian-view optical system with a 2-mm entrance pupil illuminated by a 900-W Xenon arc, instead of normal colour vision tests. The subjects had previous knowledge of the blue tinge induced after Viagra® ingestion [62], so measurements were made to isolate the S-cone response. All the subjects had statistically significant sensitivity loss when this was carried out (mild-to-moderate). The main limitation in this study was the fact that the participants were the experts. Even though the testing removed the aspect of them being aware of the blue tinge, it should have been regular patients or non-experts so that it could be a more representative study. They also did not state which test was done to determine their normal colour vision at the beginning; thus, the reliability of the participants having no prior CVD was not evident. One other disadvantage of the study was that one of the patients was aware when under the placebo and when under the actual drug; however, this showed that there were definitely visible colour changes between those categories.

Roessler et al. [63] conducted a study looking into the connection between vardenafil and ocular side effects/retinal function. They doubled the expected dosage value to 40 mg, and only one eye was tested. FM-100 hue was used one, six, and 24 hours after the drug administration as only the short-term effects were analyzed. Vardenafil did cause a temporary but significant increase in the total error score between one hour and six hours and error lines up to one hour. This is in agreement with the statement that the effect of PDE-5 inhibitors on colour vision is transient, as the FM D100 total error did not change after 24 hours. However, immediately after one hour and six hours, there was a significant change. All these studies proved that there was a significant connection between PDE-5 inhibitors and induced dyschromatopsia. The findings were consistent with the multiple studies showing induced dyschromatopsia with the use of PDE-5 inhibitors. The study done by Roessler et al. [63] should have considered the long-and short-term effects of the drug. Additionally, the study doubled the highest prescriptible future dose, which is reflective of the future use of the drug. However, it did show the potentiality of drug overdose and drug toxicity visual effects. It should have been monitored in both eyes rather than one, which was not done in this study. Acquired dyschromatopsia could have occurred in either eye; although it would have made the test longer, it would have improved the accuracy if both eyes had been observed.

Overall, there were several limitations with the studies completed for the PDE-5 inhibitors. In some of the studies, a higher dosage than normal was being taken, and an ocular toxicity reaction was was expected. The majority of patients were not monitored for a longer period, so the long-term effect was not monitored. As only the short term was looked at, the recovery may have been worse. Finally, the studies did not have a large enough population size to remove factors such as age or gender to ensure that the results were solely down to the drug usage, therefore affecting generalisability. It is difficult to compare the studies as different methods and periods of time were used to assess the CVDs acquired by the patients. Nonetheless, all the studies agreed on the fact that PDE-5 inhibitors will lead to a transient and reversible CVD, whether significant or not. The concentration of the drug did make a difference, as the higher the concentration/dosage of a PDE-5 inhibitor, the greater the likelihood of the development of a color vision defect. The most common form of acquired dyschromatopsia seen in the patients was a blue-yellow (tritanopia) defect.

## Conclusions

Overall, there is a significant connection between the use of all the drugs mentioned and acquired dyschromatopsia. They all have varying degrees of effects; the studies mentioned have confirmed some potential for dyschromatopsia to occur. For future drug development to avoid the potentiality of dyschromatopsia, it would be recommended for more investigations into varying drug dosages and concentrations, as well as changing drug structures. More needs to be done for the management of acquired CVDs; none of the studies explored solutions other than drug withdrawal, which is not always possible. There are also drugs such as CQ or HCQ where the colour vision does not always return. All of the drugs mentioned are commonly used by the global population, so any improvements to side effects, such as colour vision, would be symbolic. If acquired dyschromatopsia could be eliminated, many other associated ocular toxicity symptoms would also be reduced or removed.
